# The frequency and timing of sepsis-associated coagulopathy in the neonatal intensive care unit

**DOI:** 10.3389/fped.2024.1364725

**Published:** 2024-03-05

**Authors:** Khyzer B. Aziz, Matthew Saxonhouse, Divya Mahesh, Kathryn E. Wheeler, James L. Wynn

**Affiliations:** ^1^ Department of Pediatrics, Johns Hopkins University, Baltimore, MD, United States; ^2^ Department of Pediatrics, Wake Forest School of Medicine, Levine Children’s Hospital, Atrium Healthcare, Charlotte, NC, United States; ^3^ College of Medicine, University of Florida, Gainesville, FL, United States; ^4^ Department of Pediatrics, University of Florida, Gainesville, FL, United States

**Keywords:** neonate, sepsis, coagulation, prothrombin time, partial thromboplastin time, NICU

## Abstract

**Introduction:**

Sepsis is a common cause of morbidity and mortality in the neonatal intensive care unit (NICU). The frequency and severity of sepsis-associated coagulopathy as well as its relationship to illness severity are unclear.

**Methods:**

We performed a single-center, retrospective, observational cohort study of all infants admitted to the University of Florida Health (UF Health), level IV NICU between January 1st 2012 to March 1st 2020 to measure the frequency of sepsis-associated coagulopathy as well as its temporal relationship to critical illness in the NICU population. All clinical data in the electronic health record were extracted and deposited into an integrated data repository that was used for this work.

**Results:**

We identified 225 new sepsis episodes in 216 patients. An evaluation for sepsis-associated coagulopathy was performed in 96 (43%) episodes. Gram-negative pathogen, nSOFA score at evaluation, and mortality were greater among episodes that included a coagulopathy evaluation compared with those that did not. Abnormal coagulation results were common (271/339 evaluations; 80%) and were predominantly prothrombin times. Intervention (plasma or cryoprecipitate) followed a minority (84/271; 31%) of abnormal results, occurred in 40/96 (42%) episodes that were often associated with >1 intervention (29/40; 73%), and coincided with thrombocytopenia in 37/40 (93%) and platelet transfusion in 27/40 (68%). Shapley Additive Explanations modeling demonstrated strong predictive performance for the composite outcome of death and/or treatment for coagulopathy in neonates (f1 score 0.8, area under receiver operating characteristic curve 0.83 for those with abnormal coagulation values). The three most important features influencing the composite outcome of death or treatment for coagulopathy included administration of vasoactive medications, hematologic dysfunction assessed by the maximum nSOFA platelet score, and early sepsis (≤72 h after birth).

**Conclusions:**

A coagulopathy evaluation was performed in a minority of NICU patients with sepsis and was associated with greater illness severity and mortality. Abnormal results were common but infrequently associated with intervention, and intervention was contemporaneous with thrombocytopenia. The most important feature that influenced the composite outcome of death or treatment for coagulopathy was the administration of vasoactive-inotropic medications. These data help to identify NICU patients at risk of sepsis-associated coagulopathy.

## Introduction

Sepsis is a common cause of morbidity and mortality in the neonatal population. In adults, sepsis is defined as a life-threatening organ dysfunction caused by a dysregulated host response to infection (1). In children, sepsis-associated life-threatening organ dysfunction is operationalized using the Phoenix Sepsis Score (2). There is no consensus definition of sepsis in neonates. Sepsis-associated hematologic dysfunction manifesting as thrombocytopenia is well described and included in common sequential organ failure assessment (SOFA) scoring systems used in adults (3), children (pSOFA) (4), and neonates (nSOFA) (5). In addition to the consumption of platelets, clotting factor consumption commonly occurs with sepsis in children (41%) and adults (26%) (6, 7), is modified by the severity of illness, and increases the risk of poor outcomes including death (6, 8, 9). Other factors such as the host inflammatory response and pathogen modify the likelihood of coagulopathy (10). In 1976, Hutter and Colleagues described abnormalities in common coagulation assessments including fibrinogen, prothrombin time (PT), and partial thromboplastin time (PTT) among 14 infants with severe necrotizing enterocolitis (11). However, the frequency and severity of sepsis-associated coagulopathy in neonates, as well as the relationship of this entity to illness severity in this unique population, are unknown. Knowledge of the frequency of sepsis-associated coagulopathy reflected in commonly used tests in a large cohort, as well as the factors that place neonatal patients at risk for this entity, have potential to improve sepsis-related outcomes in this unique population.

Using a granular, single-center registry that included all data from the electronic health record (EHR) system for over a period of 9 years, we aimed to determine the following:(1) the frequency and timing of sepsis-associated coagulopathy evaluation, (2) the frequency, timing, and type of abnormal coagulation test results, (3) the frequency and timing of intervention with plasma or cryoprecipitate, and (4) the relation of sepsis-associated coagulopathy to illness severity in the neonatal intensive care unit (NICU) population.

## Methods

### Patients

This was a single-center, retrospective, observational cohort study approved by the University of Florida Health (UF Health) Institutional Review Board prior to the collection of any data. An integrated data repository (IDR) of all clinical data (structured and unstructured) in the EHR for all infants admitted to the UF Health, level IV NICU between January 1st 2012 to March 1st 2020 was created. Clinical variables that were unavailable in the IDR were collected via chart review. Demographic variables and outcomes were defined as previously reported (12–19). Maximum and hourly neonatal sequential organ failure assessment (nSOFA) scores and vasoactive-inotropic scores (VIS) were calculated as previously described (13, 19, 20).

### Definition of sepsis

An episode of sepsis was defined as a new episode of bacteremia/fungemia or meningitis with bacteria or fungus that was associated with ≥5 days of antimicrobial treatment or mortality while receiving antimicrobial treatment if duration was <5 days. Episodes were excluded if there was parenteral antimicrobial exposure within 48 h of the time of the reference culture. Coagulase negative *Staphylococcus* (CoNS) episodes were included only if there was effective antimicrobial therapy prescribed for ≥5 days or death <5 days. Infants with positive blood cultures with organisms that are frequently considered contaminants, including *Bacillus*, *Micrococcus*, and *Corynebacterium* species, were excluded.

### Coagulation evaluation

A coagulation evaluation was included if it included simultaneous measurement of at least two parameters of the following three tests: fibrinogen, PT, and PTT. Reference values measured at discrete chronologic ages where testing has been performed in this population vary by gestational age at birth and chronological age (21–24) (Supplementary Table S1). Because daily normative values for these tests from birth are not available for this population, we applied normative ranges for each coagulation measure by gestational age at birth and discrete postnatal age in a fill-forward manner (until the next tested interval where normative values have been described).

### Analytical methods

Non-parametric continuous variables were summarized as medians with quartiles (25th and 75th percentiles). Categorical variables were presented as percentages. Demographic variables were compared using Chi-square or Mann–Whitney *U* test. The nSOFA scores at discrete time intervals between multiple groups were compared using a Kruskal–Wallis test with Dunn's multiple comparisons test. Shapley Additive Explanation (SHAP) values were used to determine which variables were most important in regression modeling for patients who received treatment vs. no treatment for coagulopathy. Machine learning modeling was performed for specific variables and composite outcome for death and/or treatment to prevent over-fitting. The SHAP value is the average marginal contribution of a feature value across all possible coalitions. The threshold for statistical significance was less than 0.05 for two-sided tests. Analyses were performed using GraphPad Prism (version 10) and Python (Version 3.7; SHAP).

## Results

### Patient characteristics

Among 7,186 patients cared for in the UF NICU during the study period, we identified 910 positive blood or cerebrospinal fluid cultures in 367 patients. After applying our sepsis definition, 225 sepsis episodes in 216 patients were studied (Table 1). Episodes associated with coagulation evaluation (*n* = 96) occurred at an earlier age (13 vs. 23 days old), were more often associated with gram-negative (52% vs. 20%) and less often gram-positive (38% vs. 74%) pathogens, and showed greater nSOFA scores and greater mortality as compared with episodes without coagulation evaluation.

**Table 1:** Cohort and episode characteristics.

	No coagulation evaluation (*n* = 122 patients)	Coagulation evaluation (*n* = 94 patients)	*p*-Value^a^
Patients			
Inborn, *n* (%)	92 (75%)	79 (84)	0.12
Gestational age, median (quartiles)	31 (26, 37)	30 (26, 36)	0.78
Birth weight, median (quartiles)	1,330 (784, 2,547)	1,485 (759, 2,366)	0.82
Male, *n* (%)	67 (55)	49 (52)	0.68
Major congenital anomaly,^b^ *n* (%)	33 (27)	31 (33)	0.34
Episode characteristics^c^	*N* = 129 episodes	*N* = 96 episodes	
Prior coagulation evaluation, *n* (%)	51 (40)	42 (44)	0.53
Prior receipt of fresh frozen plasma, *n* (%)	25 (19)	15 (16)	0.47
Prior receipt of cryoprecipitate, *n* (%)	14 (11)	6 (6)	0.23
Early sepsis episode (≤72 h after birth), *n* (%)	19 (15)	14 (15)	0.98
Late sepsis episode (>72 h after birth), *n* (%)	110 (85)	82 (85)	0.98
Days old at culture, median (quartiles)	23 (8, 54)	13 (5, 33)	0.03
Gram-positive bacteria, *n* (%)	95 (74)	36 (38)	<0.0001
Gram-negative bacteria, *n* (%)	26 (20)	50 (52)	<0.0001
Polymicrobial infection, *n* (%)	6 (5)	8 (8)	0.26
Fungus, *n* (%)	2 (2)	1 (1)	0.74
Receipt of fresh frozen plasma, *n* (%)	3 (2)	36 (38)	<0.0001
Receipt of cryoprecipitate, *n* (%)	1 (1)	13 (14)	<0.0001
nSOFA score at time of culture, median (quartiles)	0 (0, 1)	2 (0, 5)	<0.0001
Maximum nSOFA score, median (quartiles)	1 (0, 5)	8 (3, 11)	<0.0001
Vasoactive-inotropic medications given, *n* (%)	10 (8)	45 (47)	<0.0001
Death with episode, *n* (%)	4 (3)	19 (20)	<0.0001
Outcomes			
Length of stay, median (quartiles)	88 (49, 119)	98 (56, 142)	0.14
28-day maximum nSOFA, median (quartiles)	6 (1, 10)	8 (4, 10)	0.048
Death, all-cause, *n* (%)	9 (7)	22 (23)	<0.001

nSOFA, neonatal sequential organ failure assessment.

^a^ Categorical variables analyzed with the chi-square test; continuous data (not normally distributed) analyzed with Mann–Whitney *U* test.

^b^ Congenital diaphragmatic hernia, gastroschisis, omphalocele, tracheoesophageal fistula, teratoma, congenital surgical heart disease (truncus arteriosus, tetralogy of Fallot, coarctation, hypoplastic left heart, hypoplastic right heart, pulmonary valve atresia, aortic valve atresia, atrioventricular canal, total anomalous pulmonary venous return, double outlet right ventricle), confirmed genetic syndrome, multiple congenital anomalies, hydrops, multicystic kidneys, posterior urethral valves with pulmonary hypoplasia.

^c^ Ten patients had two episodes.

### Pathogens

Among the 225 episodes studied, 220 were associated with bacteremia and 5 were episodes of meningitis without bacteremia. Gram-positive organisms were most common (132/225; 59%), followed by gram-negative organisms (76/225; 34%), polymicrobial infections (14/225; 6%), and minimal fungus (3/225; 1%). The most common single pathogens recovered were CoNS (68/225; 30%), *Escherichia coli* (34/225; 15%), *Staphylococcus aureus* (23/225; 10%), Group B *Streptococcus* (20/225; 9%), and *Enterococcus faecalis* (15/225; 7%). Antimicrobial resistance was common and did not differ between episodes with and without coagulopathy evaluation by pathogen class (Supplementary Table S2). Among non-CoNS episodes, 61/150 (41%) were associated with pathogens resistant to one or more common antimicrobial interventions (ampicillin, penicillin + beta-lactamase inhibitor, gentamicin, oxacillin, and third generation cephalosporin). Among episodes with recovery of non-CoNS pathogens, resistance to these common antimicrobial interventions was more common among those with a coagulopathy evaluation as compared with those without an evaluation [36/76 (47%) vs. 25/74 (34%)], but this did not reach significance (*p* = 0.10).

### Episode frequency, timing, and result of coagulation evaluation

The frequency of any coagulation assessment [PT, International normalized ratio (INR), fibrinogen, PTT] at any time among all patients cared for in the UF NICU during the study period was 23% (1,654/7,186). Coagulation evaluations during sepsis events were performed at the discretion of the clinical team. Reasons for coagulation evaluation were abstracted by chart review and included concerns for *or* documentation of bleeding (*n* = 39), thrombocytopenia (*n* = 19), sepsis (*n* = 19), preoperative or unit protocol (e.g., hypoxic-ischemic encephalopathy) (*n* = 15), or abnormal liver function tests. An analysis of available measures for study revealed that greater than 90% of sepsis episodes were associated with ≤10 total coagulation evaluations and a median of 2 evaluations per episode (IQR 1, 5). Therefore, we restricted our analyses to the first 10 coagulation measures for each episode of sepsis, which yielded 339 episode-specific coagulation evaluations performed during 96 sepsis episodes (Table 2). The median time of the first coagulation evaluation relative to the reference culture was 14 h (IQR 4, 45) and at a median age of 14 days (IQR 6, 40). Abnormal coagulation evaluation results were similarly common in the first testing of the episode (78/96, 81%) and among all testing during the episode (271/339, 80%). The most common abnormal parameter was PT (230/336, 68%) and the median time to any abnormal result was 43 h (IQR 17, 111). An abnormal coagulation evaluation result during the episode was very common and occurred in 86/96 (90%). Episode mortality among those that had a coagulation evaluation with any abnormal result was 18/86 (21%).

**Table 2:** Coagulation evaluation parameters.

Parameters during episode	Results
Coagulation evaluations per episode, median (IQR)	2 (1, 5; range 1–94)^a^
Timing of first coagulation evaluation relative to reference culture (h), median (IQR)	14 (4, 45)
Age at *first* coagulation evaluation (days)	14 (6, 40)
Frequency of any abnormal values on *first* testing (*n* = 96)	78/96 (81%)^b^
Abnormal fibrinogen (*n* = 88 measures)	Low (7/88, 8%), elevated (23/88, 26%)
Abnormal PT (*n* = 94 measures)	All prolonged (60/94, 64%)
Abnormal PTT (*n* = 90 measures)	Prolonged (14/90, 16%), fast (6/90, 7%)
Frequency of any abnormal values on *any* testing	271/339 (80%)^c^
Abnormal fibrinogen (*n* = 299 measures)	Low (14/299, 5%), elevated (58/299, 19%)
Abnormal PT (*n* = 336 measures)	All prolonged (230/336, 68%)
Abnormal PTT (*n* = 314 measures)	Prolonged (32/314, 10%), fast (21/314, 7%)
Timing of *any* abnormal result relative to reference culture (h), median (IQR) (*n* = 339)	43 (17, 111)^d^
Timing of abnormal fibrinogen (h), median (IQR)	43 (13, 118)
Timing of abnormal PT (h), median (IQR)	45 (21, 151)
Timing of abnormal PTT (h), median (IQR)	43 (13, 118)
Gestational age at birth among those with any abnormal value (weeks)	32 (26, 38)^e^

IQR, interquartile range; PT, prothrombin time; PTT, partial thromboplastin time.

^a^ Only first 10 coagulation evaluations of each episode were included in the analysis.

^b^ 14/78 (18%) had either isolated hyperfibrinogenemia, a low PTT, or both.

^c^ 239/339 (71%) had low fibrinogen, prolonged PT, or prolonged PTT.

^d^ Median timing of only abnormal (no high fib, low PTT, or combo included) 42 h (17, 103).

^e^ Median GA among only abnormal tests (no high fib, low PTT, or combo included) 32 weeks (27, 38).

### Frequency and timing of intervention and repeat testing after coagulation evaluation

Among episodes where a coagulation evaluation was performed, 40/96 (42%) were associated with an intervention (transfusion of fresh frozen plasma or cryoprecipitate). Among all coagulation evaluations, 84/271 (31%) abnormal coagulation evaluation test results were associated with subsequent intervention and 12/68 (18%) normal test results were associated with intervention (Table 3). The median timing of the first intervention was 14 h after the reference culture (IQR 4, 38). Interventions primarily occurred following the first (24/40, 60%) or second (10/40, 25%) coagulation evaluation conducted during the episode. Most episodes (29/40, 73%) were associated with >1 intervention. Repeat testing was common. Among all normal test results, 36/68 (53%) were repeated. Among abnormal test results, 214/271 (79%) were repeated.

**Table 3:** Intervention following testing.

Intervention following testing	Results
FFP^a^ or cryo^b^ given following normal test results	12/68 (18%)
FFP or cryo given following abnormal test results	84/271 (31%)^c^
Number of FFP transfusions among episodes where FFP was transfused during episode (*n* = 36), median (IQR)	3 (1, 4)
Number of cryo transfusions among episodes where cryoprecipitate was transfused during episode (*n* = 13), median (IQR)	1 (1, 2)
Episodes associated with receipt of either cryo or FFP only once	11/40 (28%)
Episodes with associated with receipt of multiple transfusions (cryo and FFP, >1 cryo, >1 FFP)	29/40 (73%)
Intervention frequency with abnormal test parameter	84/271 (31%) in 40 episodes^c^
Abnormal fibrinogen (*n* = 84)	16/84 (19%) total; 8/84 (9.5%) low; 8/84 high (9.5%)
Abnormal PT (*n* = 84)	80/84 (95%) prolonged
Abnormal PTT (*n* = 84)	16/84 (19%) total; 2/84 (2%) fast, 14/84 (17%) prolonged
Timing of first intervention (h), median (IQR).	14 (4, 38)^d^
Repeat testing performed when all parameters were normal	36/68 (53%)
Repeat testing performed when any parameter was abnormal	214/271 (79%)^e^
Platelets <150,000/µL among those with coagulation evaluation during episodes but without intervention (*n* = 56)	34/56 (61%) platelets <150,000/µL; 29/56 (52%) platelets <100,000/µL; 14/56 (25%) platelets <50,000/µL
Platelets <150,000/µL among those with coagulation evaluation during episodes and intervention (*n* = 40)	37/40 (93%) platelets <150,000/µL; 29/40 (73%) platelets <100,000/µL; 19/40 (48%) platelets <50,000/µL
Platelet transfusion among those episodes with coagulation evaluation (*n* = 96)	48/96 (50%) all episodes; 21/56 (38%) no FFP/cryo received; 27/40 (68%) FFP/cryo received

IQR, interquartile range; PT, prothrombin time; PTT, partial thromboplastin time.

^a^ Fresh frozen plasma.

^b^ Cryoprecipitate.

^c^ 81/239 (34%) excluding high fibrinogen, low PTT, or combo.

^d^ Specimen 1: 24/40 episodes, Specimen 2: 10/40 episodes, Specimen 3: 3/40 episodes, Specimen 6, 9, and >10 each had 1.

^e^ 199/239 repeated excluding high fibrinogen, low PTT, or combination of high fibrinogen and low PTT.

### Frequency of concurrent abnormal platelet counts

Among the 56 episodes where a coagulation evaluation was performed without associated intervention, 34 (61%) were associated with mild thrombocytopenia (<150,000/µL), 29 (52%) with moderate thrombocytopenia (<100,000/µL), and 14 (25%) with severe thrombocytopenia (<50,000/µL) (Table 3). Among the 40 episodes where coagulation evaluation was performed with associated intervention, 37 (93%) were associated with mild thrombocytopenia, 29 (73%) with moderate thrombocytopenia, and 19 (48%) with severe thrombocytopenia. Out of the 96 episodes where a coagulation evaluation was performed (regardless of intervention), 48 episodes were associated with ≥1 platelet transfusions (27/40 (68%) episodes with intervention; 21/56 (38%) episodes without intervention).

### nSOFA trajectories

**Figure 1:**
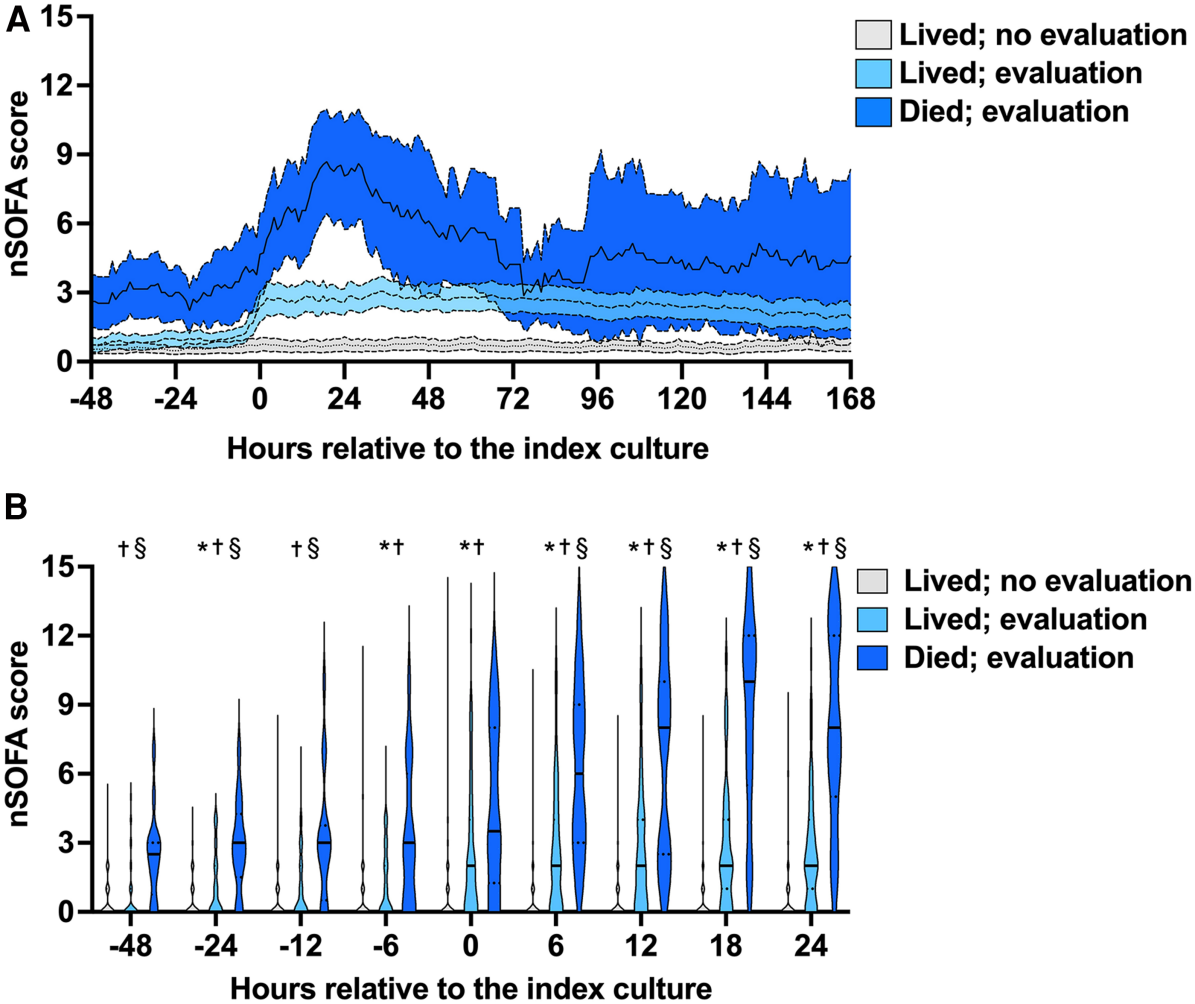
Neonatal sequential organ failure assessment (nSOFA) score trajectories and timed comparisons. (**A**) Consecutive q1-h nSOFA score mean (internal line) and 95% confidence intervals (surrounding band) during episodes with (lived, *n* = 77; died *n* = 19) and without (lived, *n* = 125) a coagulation evaluation (4 patients without a coagulation evaluation died and are not shown). (**B**) nSOFA scores at multiple discrete time points relative to the index blood culture (time 0) among the same groups. Violin plots show median (solid line) and quartiles (dotted line). Comparisons were made by Kruskal–Wallis with Dunn's multiple comparisons test. Maximum possible nSOFA score was 15 for any time point. *No evaluation vs. evaluated and lived; *p* ≤ 0.03; ^†^no evaluation vs. evaluated and died; *p* ≤ 0.0004; ^§^evaluated and lived vs. evaluated and died *p* ≤ 0.005.

We examined nSOFA scores during episodes with (lived, *n* = 77; died *n* = 19) and without (lived, *n* = 125) a coagulation evaluation at hourly resolution (Figure 1A) and compared scores at multiple discrete time points (Figure 1B) relative to the reference blood culture. Median nSOFA scores were 0 for survivors that did not have a coagulation evaluation at all nine discrete time points we evaluated (IQR for all nine time points: 0, 1). Survivors that had a coagulation evaluation also demonstrated a median nSOFA of 0 prior to evaluation (range of all IQR: 0, 2) but increased to 2 at evaluation (IQR 0, 4) and remained 2 at all future timepoints (range IQR 0–1, 4). Episodes with a coagulation evaluation that ended in death demonstrated a median nSOFA of 3 prior to evaluation (range IQR 0–2, 3–6) that increased to 4 (IQR 2, 8) at evaluation, 6 (IQR 3, 9) at 6 h, 8 (IQR 3, 10) at 12 h, 10 (IQR 6, 12) at 18 h, and 8 (IQR 5, 12) at 24 h.

### Regression modeling

**Figure 2:**
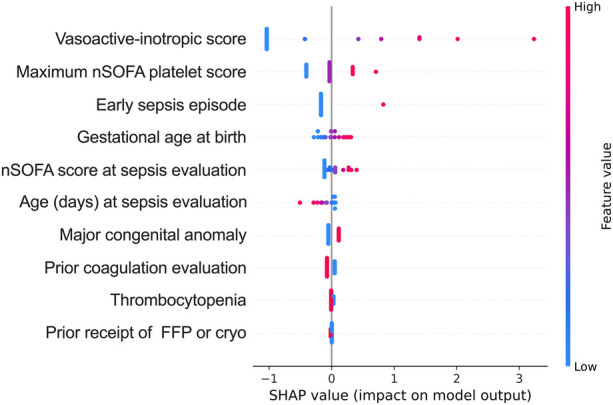
Shapley Additive explanation (SHAP) value plots provide a hierarchical organization of various features included in the model to predict the likelihood of death and/or treatment in the patients with abnormal coagulation values. Red and blue dots represent higher and lower values of the feature; maximum nSOFA platelet score (during episode); early sepsis episode (sepsis ≤72 h after birth); nSOFA, neonatal sequential organ failure assessment.

Logistic regression modeling and SHAP analysis were conducted to evaluate the significance of clinical variables and abnormal coagulation values in predicting outcomes (Supplementary Table S3 and Figure 2). The SHAP modeling demonstrated strong predictive performance for the composite outcome of death and/or treatment for coagulopathy in neonates (f1 score 0.8, area under receiver operating characteristic curve 0.83 for those with abnormal coagulation values). The three most important features influencing death and/or treatment for coagulopathy included administration of vasoactive medications, quantified by the VIS, hematologic dysfunction assessed by the maximum nSOFA platelet score, and early sepsis (within ≤72 h after birth).

## Discussion

This is the first comprehensive study of coagulopathy specific to common clotting parameters among patients with sepsis in the NICU population. A coagulopathy evaluation was performed in a minority of NICU patients with sepsis. Gram-negative pathogen, nSOFA score at evaluation, and mortality were greater and chronologic age at sepsis evaluation was lower among episodes that included a coagulopathy evaluation compared with those that did not. Abnormal results were common but infrequently associated with intervention, and intervention was nearly always associated with thrombocytopenia. These data may help to identify and guide treatment for future NICU patients at high risk of sepsis-associated coagulopathy.

Our findings aligned with well-described clinical risks and characteristics for coagulopathy in other populations including gram-negative pathogens, high severity of illness including shock, and coexistence of thrombocytopenia (10). A lower severity of illness and mortality frequency among those that did not have a coagulation evaluation compared with those that did in our study mirrored results in a large pediatric population evaluated for suspected sepsis in the emergency room setting (9). Among all sepsis episodes, intervention with either fresh frozen plasma or cryoprecipitate for any reason was documented in 43/225 (19%) episodes. Among patients not evaluated for coagulopathy, use of fresh frozen plasma or cryoprecipitate as well as mortality was rare and does not support that significant coagulopathy was present but missed. There are several potential reasons for neonates to exhibit reduced coagulopathy. One potential contributor to the frequency of coagulopathy in neonates is a unique host response to sepsis including a reduced inflammatory response as compared with the other age groups (25, 26). In an analysis of publicly available genome-wide expression microarrays of whole blood samples from humans of all ages with sepsis, neonates manifested a reduced pro-inflammatory response compared with other age groups (27). Furthermore, most sepsis events in neonates are associated with gram-positive bacterial recovery from blood, with CoNS being the most commonly identified gram-positive pathogen (28). The inflammatory response associated with CoNS, and thus the instigation of aberrant clotting, may be attenuated (29).

Concurrent thrombocytopenia was nearly ubiquitous among those with abnormal clotting parameters that received treatment. Coagulation dysfunction criteria in critically ill children were recently proposed following systematic review as part of the PODIUM Consensus Conference (30). Importantly, this comprehensive work was not intended for the preterm or NICU population. In addition to thrombocytopenia (<100,000 platelets/µL), the coagulation dysfunction criteria identified included INR (>1.5), fibrinogen (<150 mg/dL), and D-dimer (>10 times the upper limit of normal). The published parameters we used to guide normative coagulation values in our study reported PT rather than INR values, therefore we focused on PT. As expected, we found abnormally prolonged PT measures in most patients evaluated, which is consistent with known reduced activities of vitamin K-dependent factors in this population (31). Despite the high frequency of abnormal PT measures, most of these abnormal values were not associated with intervention. This finding suggests that the values returned were interpreted by the clinical team as acceptable, despite being considered abnormal based on the available normative data. Furthermore, perhaps because of the historical difficulty in interpretation in neonates (32), D-Dimer is neither a test routinely conducted on the NICU population nor sufficiently measured in this cohort to facilitate meaningful study (51 D-dimer measures in 22 patients among 339 coagulation tests studied). Rotational thromboelastometry, which evaluates phases of the clotting process, showed utility to identify those at the highest risk of mortality when routinely sent among a cohort of neonates with sepsis (33). As developmental normative values are being characterized, this testing may represent an additional or alternative way to measure the effectiveness of clotting in neonates with sepsis (34).

Less than half (96/225) of the patients in this near decade cohort received a coagulopathy evaluation at any time during the sepsis episode. Based on our collective clinical experiences at multiple academic institutions over several decades, we speculate the volume of blood required for coagulation testing (1.8 mL at UF) diminishes the frequency of routine coagulation evaluations in this population. Because the coagulation evaluations in this study were clinician-driven and not performed in all patients, we cannot accurately determine the frequency of coagulation abnormalities. In our cohort, 28% of infants with gram-positive bacteremia were evaluated, and 38% of those received cryoprecipitate or fresh frozen plasma. By contrast, 66% of infants with gram-negative bacteremia were evaluated for coagulopathy (2.4-fold over gram-positive) and 49% (1.3-fold over gram-positive) of those received cryoprecipitate or fresh frozen plasma. Based on our results, we suggest that coagulation evaluation be considered in all infants with presumed or confirmed sepsis and especially those with administration of vasoactive or inotropic medications. The data presented in our study does not allow for speculation regarding healthcare provider decisions to: (1) conduct assessments for coagulopathy, or (2) determine the rationale behind the treatment administered for coagulopathy.

## Limitations

We acknowledge the true incidence of coagulopathy with sepsis in neonates cannot be derived from these data because the presence and timing of coagulopathy evaluation was not universal but rather was performed at the discretion of clinical providers. However, this is the largest and most comprehensive study of coagulopathy among patients with sepsis in the NICU population. Additional limitations in generalizability inherent to the single-center and retrospective nature of the study apply. Normal coagulation parameters by chronologic age, especially in the most immature infants, are unclear or in some cases entirely unavailable. Guidelines for when to measure clotting parameters and what results should guide treatment in this population are not standardized. Our study represents a first step toward addressing these many knowledge gaps and challenges.

## Conclusions

In this cohort, only a minority of NICU patients with sepsis had a coagulation evaluation. Abnormal results were common but infrequently associated with intervention, and intervention was nearly always associated with thrombocytopenia. These data may help to identify future NICU patients at high risk of sepsis-associated coagulopathy.

## Data Availability

The raw data supporting the conclusions of this article will be made available by the authors, without undue reservation.

## References

[1] Singer M Deutschman CS Seymour CW Shankar-Hari M Annane D Bauer M The third international consensus definitions for sepsis and septic shock (sepsis-3). JAMA. (2016) 315:801–10. 10.1001/jama.2016.0287 26903338 PMC4968574

[2] Schlapbach LJ Watson RS Sorce LR Argent AC Menon K Hall MW International consensus criteria for pediatric sepsis and septic shock. JAMA. (2024). 10.1001/jama.2024.0179 PMC10900966 38245889

[3] Vincent JL Moreno R Takala J Willatts S De Mendonca A Bruining H The SOFA (Sepsis-related Organ Failure Assessment) score to describe organ dysfunction/failure. On behalf of the working group on sepsis-related problems of the European Society of Intensive Care Medicine. Intensive Care Med. (1996) 22:707–10. 10.1007/BF01709751 8844239

[4] Matics TJ Sanchez-Pinto LN. Adaptation and validation of a pediatric sequential organ failure assessment score and evaluation of the sepsis-3 definitions in critically ill children. JAMA Pediatr. (2017) 171:e172352. 10.1001/jamapediatrics.2017.2352 28783810 PMC6583375

[5] Wynn JL Polin RA. A neonatal sequential organ failure assessment score predicts mortality to late-onset sepsis in preterm very low birth weight infants. Pediatr Res. (2020) 88:85–90. 10.1038/s41390-019-0517-2 31394566 PMC7007331

[6] Loberger JM Aban IB Prabhakaran P. Exploration of sepsis-associated coagulopathy severity and pediatric septic shock outcomes. J Pediatr Intensive Care. (2021) 10:38–44. 10.1055/s-0040-1713436 33585060 PMC7870337

[7] Schmoch T Mohnle P Weigand MA Briegel J Bauer M Bloos F The prevalence of sepsis-induced coagulopathy in patients with sepsis—a secondary analysis of two German multicenter randomized controlled trials. Ann Intensive Care. (2023) 13:3. 10.1186/s13613-022-01093-7 36635426 PMC9837358

[8] Lyons PG Micek ST Hampton N Kollef MH. Sepsis-associated coagulopathy severity predicts hospital mortality. Crit Care Med. (2018) 46:736–42. 10.1097/CCM.0000000000002997 29373360

[9] Slatnick LR Thornhill D Deakyne Davies SJ Ford JB Scott HF Manco-Johnson MJ Disseminated intravascular coagulation is an independent predictor of adverse outcomes in children in the emergency department with suspected sepsis. J Pediatr. (2020) 225:198–206.e2. 10.1016/j.jpeds.2020.06.022 32553867 PMC7529972

[10] Levi M Van Der Poll T. Coagulation and sepsis. Thromb Res. (2017) 149:38–44. 10.1016/j.thromres.2016.11.007 27886531

[11] Hutter JJ Jr Hathaway WE Wayne ER. Hematologic abnormalities in severe neonatal necrotizing enterocolitis. J Pediatr. (1976) 88:1026–31. 10.1016/S0022-3476(76)81069-4 1271173

[12] Alexandesr GR Himes JH Kaufman RB Mor J Kogan M. A United States national reference for fetal growth. Obstet Gynecol. (1996) 87:163–8. 10.1016/0029-7844(95)00386-X 8559516

[13] Aziz KB Schles EM Makker K Wynn JL. Frequency of acute kidney injury and association with mortality among extremely preterm infants. JAMA Netw Open. (2022) 5:e2246327. 10.1001/jamanetworkopen.2022.46327 36512358 PMC9856227

[14] Jensen EA Dysart K Gantz MG Mcdonald S Bamat NA Keszler M The diagnosis of bronchopulmonary dysplasia in very preterm infants. An evidence-based approach. Am J Respir Crit Care Med. (2019) 200:751–9. 10.1164/rccm.201812-2348OC 30995069 PMC6775872

[15] Lavilla OC Aziz KB Lure AC Gipson D De La Cruz D Wynn JL. Hourly kinetics of critical organ dysfunction in extremely preterm infants. Am J Respir Crit Care Med. (2022) 205:75–87. 10.1164/rccm.202106-1359OC 34550843 PMC8865589

[16] Papile LA Burstein J Burstein R Koffler H. Incidence and evolution of subependymal and intraventricular hemorrhage: a study of infants with birth weights less than 1,500 gm. J Pediatr. (1978) 92:529–34. 10.1016/S0022-3476(78)80282-0 305471

[17] Walsh MC Kliegman RM. Necrotizing enterocolitis: treatment based on staging criteria. Pediatr Clin North Am. (1986) 33:179–201. 10.1016/S0031-3955(16)34975-6 3081865 PMC7131118

[18] Stoll BJ Hansen NI Bell EF Walsh MC Carlo WA Shankaran S Trends in care practices, morbidity, and mortality of extremely preterm neonates, 1993–2012. JAMA. (2015) 314:1039–51. 10.1001/jama.2015.10244 26348753 PMC4787615

[19] Yeo KT Goh GL Park WY Wynn JL Aziz KB. Evaluation of the neonatal sequential organ failure assessment and mortality risk in neonates with early-onset infection. Neonatology. (2023) 120:796–800. 10.1159/000533467 37757759 PMC13202496

[20] Wynn JL Mayampurath A Carey K Slattery S Andrews B Sanchez-Pinto LN. Multicenter validation of the neonatal sequential organ failure assessment score for prognosis in the neonatal intensive care unit. J Pediatr. (2021) 236:297–300.e1. 10.1016/j.jpeds.2021.05.037 34022247 PMC9045002

[21] Andrew M Paes B Milner R Johnston M Mitchell L Tollefsen DM Development of the human coagulation system in the healthy premature infant. Blood. (1988) 72:1651–7. 10.1182/blood.V72.5.1651.1651 3179444

[22] Andrew M Paes B Milner R Johnston M Mitchell L Tollefsen DM Development of the human coagulation system in the full-term infant. Blood. (1987) 70:165–72. 10.1182/blood.V70.1.165.165 3593964

[23] Neary E Mccallion N Kevane B Cotter M Egan K Regan I Coagulation indices in very preterm infants from cord blood and postnatal samples. J Thromb Haemost. (2015) 13:2021–30. 10.1111/jth.13130 26334448

[24] Neary E Okafor I Al-Awaysheh F Kirkham C Sheehan K Mooney C Laboratory coagulation parameters in extremely premature infants born earlier than 27 gestational weeks upon admission to a neonatal intensive care unit. Neonatology. (2013) 104:222–7. 10.1159/000353366 24030102

[25] Wynn J Cornell TT Wong HR Shanley TP Wheeler DS. The host response to sepsis and developmental impact. Pediatrics. (2010) 125:1031–41. 10.1542/peds.2009-3301 20421258 PMC2894560

[26] Wynn JL Cvijanovich NZ Allen GL Thomas NJ Freishtat RJ Anas N The influence of developmental age on the early transcriptomic response of children with septic shock. Mol Med. (2011) 17:1146–56. 10.2119/molmed.2011.00169 21738952 PMC3321808

[27] Raymond SL Lopez MC Baker HV Larson SD Efron PA Sweeney TE Unique transcriptomic response to sepsis is observed among patients of different age groups. PLoS One. (2017) 12(9):e0184159. 10.1371/journal.pone.0184159 28886074 PMC5590890

[28] Flannery DD Edwards EM Coggins SA Horbar JD Puopolo KM. Late-onset sepsis among very preterm infants. Pediatrics. (2022) 150(6):e2022058813. 10.1542/peds.2022-058813 36366916 PMC11151779

[29] Klingenberg C Aarag E Ronnestad A Sollid JE Abrahamsen TG Kjeldsen G Coagulase-negative staphylococcal sepsis in neonates. Association between antibiotic resistance, biofilm formation and the host inflammatory response. Pediatr Infect Dis J. (2005) 24:817–22. 10.1097/01.inf.0000176735.20008.cd 16148849

[30] Faustino EVS Karam O Parker RI Hanson SJ Brandao LR Monagle P Coagulation dysfunction criteria in critically ill children: the PODIUM consensus conference. Pediatrics. (2022) 149:S79–83. 10.1542/peds.2021-052888l 34970670

[31] Araki S Shirahata A. Vitamin K deficiency bleeding in infancy. Nutrients. (2020) 12(3):780. 10.3390/nu12030780 32187975 PMC7146284

[32] Hudson IR Gibson BE Brownlie J Holland BM Turner TL Webber RG. Increased concentrations of D-dimers in newborn infants. Arch Dis Child. (1990) 65:383–4. 10.1136/adc.65.4_Spec_No.383 2337364 PMC1590146

[33] Sokou R Tsantes AG Lampridou M Tsante KA Houhoula D Piovani D Thromboelastometry and prediction of in-hospital mortality in neonates with sepsis. Int J Lab Hematol. (2024) 46(1):113–9. 10.1111/ijlh.14165 37641388

[34] Cannata G Mariotti Zani E Argentiero A Caminiti C Perrone S Esposito S. TEG® and ROTEM® Traces: clinical applications of viscoelastic coagulation monitoring in neonatal intensive care unit. Diagnostics (Basel). (2021) 11(9):1642. 10.3390/diagnostics11091642 34573982 PMC8465234

