# Supplementary material for: The frequency and timing of sepsis-associated coagulopathy in the neonatal intensive care unit
Source: Front Pediatr. 2024 Mar 5;12:1364725. doi: 10.3389/fped.2024.1364725 (PMC10948397; doi:10.3389/fped.2024.1364725)
Supplement: Supplementary file 1 [file Datasheet1.pdf]

Supplemental table 1. Normative values by birth gestational age and chronologic age

Published values	Day 1	Day 3-5	Day 15-30	Day 90	Day 180
Fill forward	Day 1-2	Day 3-14	Day 15-89	Day 90-179	Day 180+
Term ≥37 weeks at birth					
Prothrombin time	13 (10.1-15.9)	12.4 (10-15.3)	11.8 (10-14.2)	11.9 (10-14.2)	12.3 (10.7-13.9)
Partial thromboplastin time	42.9 (31.3-54.5)	42.6 (25.4-59.8)	40.4 (25.6-55.2)	37.1 (24.1-50.1)	35.5 (28.1-42.9)
Fibrinogen	283 (167-399)	312 (162-462)	270 (162-378)	243 (150-379)	251 (150-387)
Preterm 30-36 weeks at birth					
Prothrombin time	13 (10.6-16.2)	12.5 (10-15.3)	11.8 (10-13.6)	12.3 (10-14.6)	12.5 (10-15)
Partial thromboplastin time	53.6 (27.5-79.4)	50.5 (26.9-74.1)	44.7 (26.9-62.5)	39.5 (28.3-50.7)	37.5 (21.7-53.3)
Fibrinogen	243 (150-373)	280 (160-418)	254 (150-414)	246 (150-352)	228 (150-360)
Very preterm 23-29 weeks at birth					
Prothrombin time	17.5 (12.7-26.6)	13.7 (11.6-16.2)	12 (11.2-16.2) →	→	→
Partial thromboplastin time	78.7 (48.7-134.3)	49.3 (35.3-68.3)	47.4 (31.9-68) →	→	→
Fibrinogen	140 (72-380) →	→	→	→	→

Data represent mean and 95% confidence intervals

Supplemental table 2. Antimicrobial resistance among isolated pathogens

	AMR: All episodes (n = 225)	AMR: No coagulation evaluation (n = 129)	AMR: Coagulation evaluation (n = 96)	p-value
All pathogens	175/225 (78%)	102/129 (79%)	73/96 (76%)	0.63
Non-CoNS pathogens	101/150 (67%)	48/74 (65%)	53/76 (70%)	0.60
Gram-negative	64/76 (84%)	23/26 (88%)	41/50 (82%)	0.53
Gram-negative mixed	1/1 (100%)	0/0 (0%)	1/1 (100%)	*
Gram-negative;Gram-positive mix**	4/6 (67%)	1/1 (100%)	3/5 (60%)	*
Gram-positive	31/64 (49%)	23/45 (51%)	8/19 (42%)	0.59
CoNS	68/68 (100%)	51/51 (100%)	17/17 (100%)	1.0
Gram-positive mix***	7/7 (100%)	4/4 (100%)	3/3 (100%)	1.0
Candida sps	0/3 (0%)	0/2 (0%)	0/1 (0%)	*

* - cannot compare groups with <3 samples

** - none of the Gram-negative;Gram-positive mix included CoNS

*** - all Gram-positive mix included CoNS

Supplemental table 3. Characteristics of episodes with coagulation evaluation

Episode characteristics	No treatment (N = 56)	Treatment (N = 40)	p-value†
Inborn, n (%)	50 (89%)	32 (80%)	0.20
Gestational age (weeks), median (quartiles)	30 (26, 36)	30 (26, 36)	0.66
Birth weight (g) , median (quartiles)	1507 (851, 2422)	1467 (699, 2538)	0.79
Male, n (%)	29 (52%)	22 (55%)	0.76
Major congenital anomaly*, n (%)	19 (34%)	13 (33%)	0.88
Prior coagulation evaluation, n (%)	25 (45%)	17 (43%)	0.83
Prior receipt of fresh frozen plasma or cryoprecipitate, n (%)	9 (16%)	8 (20%)	0.62
Early sepsis episode (≤72 hours after birth), n (%)	5 (9%)	9 (23%)	0.06
Late sepsis episode (>72 hours after birth), n (%)	51 (91%)	31 (78%)	0.06
Days old at culture, median (quartiles)	15 (7, 55)	8 (3, 24)	0.04
Gram-positive bacteria, n (%)	24 (43%)	15 (38%)	0.60
Gram-negative bacteria, n (%)	27 (48%)	24 (60%)	0.25
Polymicrobial or fungal, n (%)	5 (9%)	1 (3%)	0.20
nSOFA score at time of culture, median (quartiles)	2 (0, 4)	2 (0, 6)	0.47
Vasoactive-inotropic medications given, n (%)	13 (23%)	32 (80%)	<0.0001
Thrombocytopenia (<150,000/μL), n (%)	36 (64%)	37 (93%)	0.001
Death with episode, n (%)	5 (9%)	14 (35%)	0.002

nSOFA, neonatal sequential organ failure assessment

† Categorical variables analyzed with the chi-square test; continuous data (not normally distributed) analyzed with Mann-Whitney U test.

* - congenital diaphragmatic hernia, gastroschisis, omphalocele, tracheoesophageal fistula, teratoma, congenital surgical heart disease (*truncus arteriosus, tetralogy of Fallot, coarctation, hypoplastic left heart, hypoplastic right heart, pulmonary valve atresia, aortic valve atresia, atrioventricular canal, total anomalous pulmonary venous return, double outlet right ventricle*), confirmed genetic syndrome, multiple congenital anomalies, hydrops, multi-cystic kidneys, posterior urethral valves with pulmonary hypoplasia.
